# Long-term physical performance trends across the five confederations: Evidence from the 19th to 22nd FIFA World Cup editions (2010–2022)

**DOI:** 10.1371/journal.pone.0354205

**Published:** 2026-07-23

**Authors:** Xiao Yang, Liangzhu Feng

**Affiliations:** 1 School of Physical Education, Weinan Normal University, Weinan, China; 2 School of Physical Education & Sports Science, South China Normal University, Guangzhou, China; Universiti Malaya, MALAYSIA

## Abstract

**Background:**

Physical performance demands in elite international football have evolved substantially in recent decades, yet longitudinal comparisons across FIFA confederations remain limited.

**Objective:**

This study examined long-term changes in physical performance demands among outfield players from five confederations (UEFA, CONMEBOL, CONCACAF, CAF, and AFC) using tracking data from 225 matches at the 2010–2022 FIFA World Cups.

**Methods:**

Generalised mixed linear models were used to estimate changes in total distance, top speed, sprint (>25 km·h ⁻ ¹), low-intensity running distance (<15 km·h ⁻ ¹), and high-intensity running distance (≥15 km·h ⁻ ¹), with confederation as the main factor and match status, location, and team and opponent strength controlled.

**Results:**

Across confederations, substantial increases were observed in top speed, sprint, and low-intensity running distance from 2010 to 2022, indicating a uniform rise in speed-related demands. In contrast, changes in total distance and high-intensity running distance were confederation-specific. Total distance increased between 2018 and 2022 in AFC, CONCACAF, and UEFA, but showed no substantial change in CONMEBOL. High-intensity running distance declined in AFC, CONMEBOL, and UEFA, while changes in CAF were trivial.

**Conclusion:**

These findings indicate that speed-related demands have increased globally, whereas total and high-intensity running demands remain region-specific across FIFA World Cup editions.

## Introduction

Over the last few decades, the physical demands placed on elite football players have increased markedly, in parallel with changes in tactics, training methodologies and competition structures [1]. Early match running performance analyses already suggested that contemporary match play imposes greater physical demands than in earlier periods, with players repeatedly performing varied locomotor activities over 90 minutes and beyond [2]. The development of computer-aided motion analysis and, subsequently, fully automated multi-camera and optical tracking systems has profoundly advanced the monitoring of players’ external load profiles, enabling detailed quantification of distances covered across distinct speed zones and the frequency of high-intensity efforts in professional and international football [3].

A substantial body of research has since described the physical characteristics of elite football players at domestic and international levels. Foundational studies using systems such as Amisco Pro® provided detailed profiles of positional match demands and work intensities in top European competitions, demonstrating clear differences in total distance and high-intensity running between positions and contextual situations [4]. Subsequent work has examined how factors such as match outcome, competition level and tactical roles influence the distribution of running distances and high-intensity activity [5]. These developments laid the methodological foundations for more recent large-scale analyses of elite tournaments.

The FIFA World Cup, as the highest-level international tournament, has naturally become a focal point for such analyses. Studies of match running performance have examined overall running demands across World Cup editions as well as specific periods of the game, for example the physical and technical demands of extra time in the 2018 tournament [6,7]. Other investigations of the 2018 FIFA World Cup have linked technical and physical match performance to different playing styles, highlighting how tactical approaches may shape running outputs [8]. More recently, FIFA-endorsed work on the 2022 World Cup in Qatar has “set the benchmark” by contextualising team and positional running demands using enhanced optical tracking and standardised speed zones, thereby detailing contemporary high-intensity profiles at international level [9].

In parallel, researchers have begun to explore whether match running performance differs among continental confederations. Using data from the 2018 FIFA World Cup, Tuo et al. reported that UEFA and CONMEBOL players exhibited similar running profiles, whereas players from AFC, CAF and CONCACAF covered less total distance and less jogging or low-speed running, but spent more time walking, despite generally similar high-intensity running and sprint metrics across confederations [10]. More recent analyses of the 2022 World Cup have compared physical, technical and tactical indicators between confederations and ranking groups, reinforcing the relevance of confederation-specific perspectives [11]. Together, these studies suggest that continental context may modulate how teams express the global trend towards increased physical intensity.

Despite these advances, some aspects of World Cup match analysis research remain relatively unexplored. First, most work has focused on a single tournament (e.g., 2014, 2018 or 2022) or on pooled samples, providing limited information on long-term temporal trends across multiple World Cup cycles [6]. Second, although confederation differences have been examined for the 2018 edition [10], there has been little systematic analysis of whether the evolution of match running performance from 2010 to 2022 has followed similar or divergent trajectories across all five FIFA confederations (AFC, CAF, CONCACAF, CONMEBOL and UEFA). Third, previous work has only rarely applied the same set of speed-zone definitions and analytical procedures across several editions when describing confederation-level performance.

To address these gaps, the present study analysed match running performance data from the 19th to 22nd FIFA World Cups (2010–2022). Because different running-speed classifications were used across these tournaments, all tracking data were regrouped into a unified set of intensity bands: all running at speeds below 15 km/h was classified as low-intensity running distance (LIRD), and all running at speeds ≥15 km/h (covering the high, very-high and sprint zones) was classified as high-intensity running distance (HIRD). Based on these unified intensity bands, five physical performance parameters that were comparable across editions were defined: total distance (TD), top speed (TopS), number of sprints (Sprint, defined as efforts at >25 km/h), LIRD and HIRD. The aims of the study were to (i) describe temporal changes in these running performance parameters within each confederation and (ii) identify common patterns and confederation-specific differences in the evolution of physical match demands across four consecutive World Cup editions.

## Methods

### Sample and variables

Physical performance statistics of 3,454 players from 225 matches across the 2010–2022 FIFA World Cups were analysed (22 extra-time fixtures and 9 matches with missing data were excluded from the original 256). Original data were collected by a semi-automatic computerized video tracking system, Amisco Pro®, whose working process, accuracy, validity and reliability have been discussed in detail in prior studies [12–14]. In line with the unified speed-zone definitions, five physical performance-related parameters were chosen as dependent variables in the analysis. The grouping and definitions of these variables are listed in Table 1. Five FIFA confederations ((Europe (UEFA), South America (CONMEBOL), North/Central America and Caribbean (CONCACAF), Africa (AFC) and Asia (CFA)) were chosen as the main predictor variable, meanwhile, other four situational variables (match status, match location, team strength and opponent strength) were added as predictor variables as well. Ethical approval was not required for this study because it was based exclusively on publicly available, anonymized data.

**Table 1:** Selected physical performance-related parameters (dependent variables).

Variable	Definition
TD (m)	total distance covered in a match by all the players of a team.
TopS(km/h)	Highest speed attained during playing time
Sprint(counts)	sprint (speed over 25 km/h) distance achieved by all the players of a team in a match.
LIRD(m)	distance covered at low-speed-running (speed below 15 km/h) in a match by all the players of a team.
HIRD(m)	Distance at ≥15 km/h (high + very high + sprint)

### Procedure and statistical analysis

The generalised mixed linear modelling was realized with Proc Glimmix in the University Edition of Statistical Analysis System (version SAS Studio 3.6). Separate Poisson regressions were run in the model taking the value of each of the 5 physical performance-related parameters as the dependent variable. Random effect for team identity and match identity was used to account for repeated measurement on the teams and matches. The fixed effects estimated the effects of the four predictor variables (match period, match status, team strength and opponent strength). Match period and match status were included as nominal variables. Match period was with 2 levels (named 1 and 2, stands for the group stage and the knockout stage). Match result was with three levels (named 3, 1, and 0, stands for winning, drawing and losing). The effect of team strength and opponent strength was estimated by including the difference in the log of the end-of-season ranks as a predictor.

The established models can estimate the mean change value of each of the physical performance-related parameters across the 2010–2022 FIFA World Cups, controlling the effects of match status, match location, team strength and opponent strength. Uncertainty in the true change was evaluated using the non-clinical magnitude-based inference [15] as implemented in the spreadsheet accompanying the package of materials for generalised mixed modelling with SAS Studio [16]. Observed magnitudes and their confidence limits were expressed in standardized units, whereby the estimated mean change was divided by the observed between-match standard deviation (SD) of the first half derived from the mixed model, and then evaluated qualitatively with the following scale: < 0.2 trivial, 0.2–0.6 small, 0.6–1.2 moderate, 1.2–2.0 large, > 2.0 very large. Effects were deemed clear if the 99% confidence interval did not include positive and negative substantial values simultaneously. Clear effects were reported with a qualitative likelihood that the true change was either substantial or trivial (whichever probability was greater) using the following scale: < 0.5% most unlikely, 0.5–5% very unlikely, 5–25% unlikely, 25–75% possibly, 75–95% likely, 95–99.5% very likely, > 99.5% most likely. To minimize statistical error, only effects with a probability greater than 95% were considered in the analysis and discussion.

## Result

**Fig 1:**
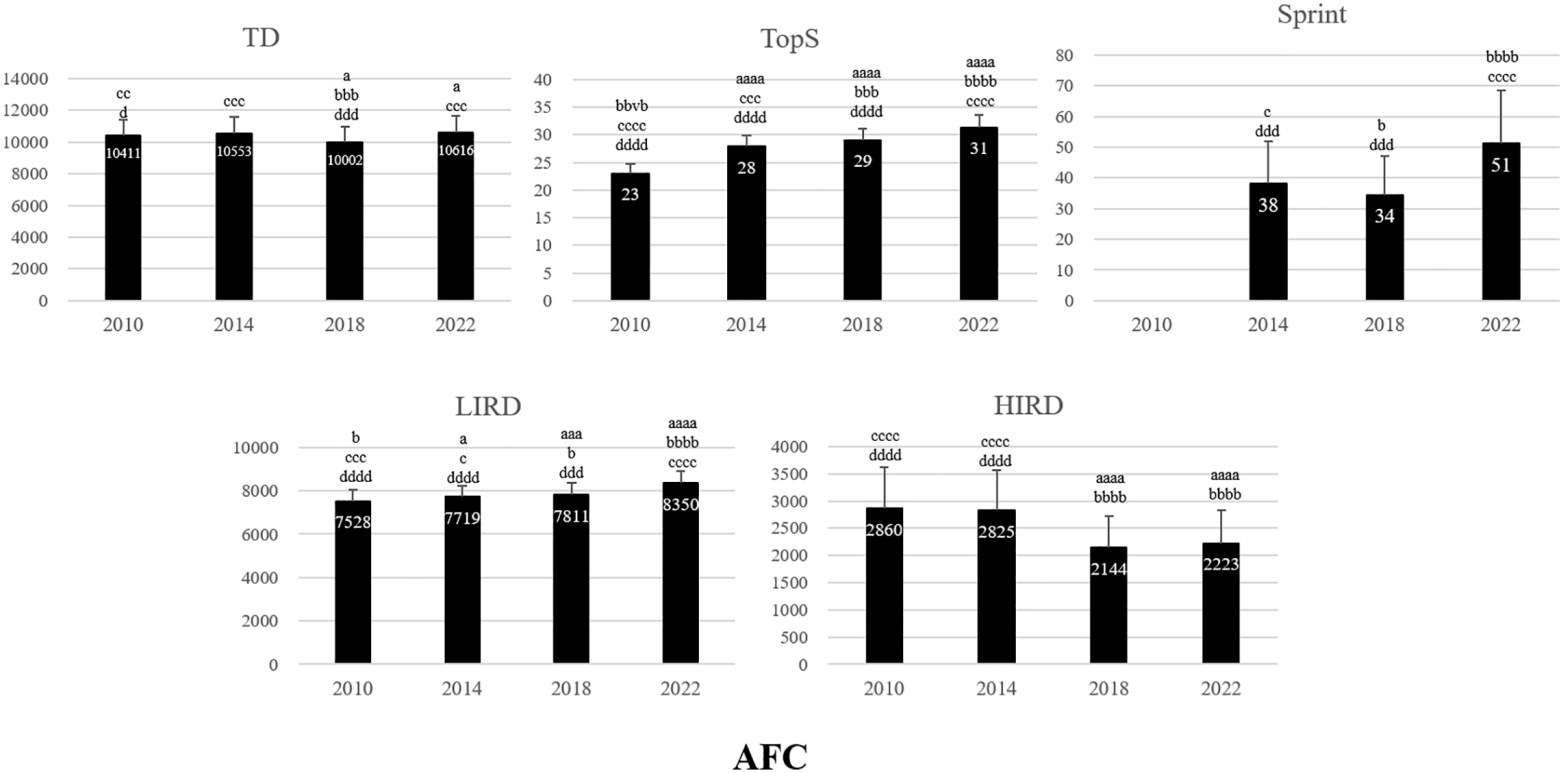
Changes in the values of physical parameters for outfield players in AFC across the 2010–2022 FIFA World Cup. Superscript letters “a–d” denote statistically significant differences relative to the 19th, 20th, 21st, and 22nd cohorts, respectively. The number of letters (1–4) indicates the level of significance: one letter, possible; two letters, likely; three letters, very likely. four letters, most likely.

**Fig 2:**
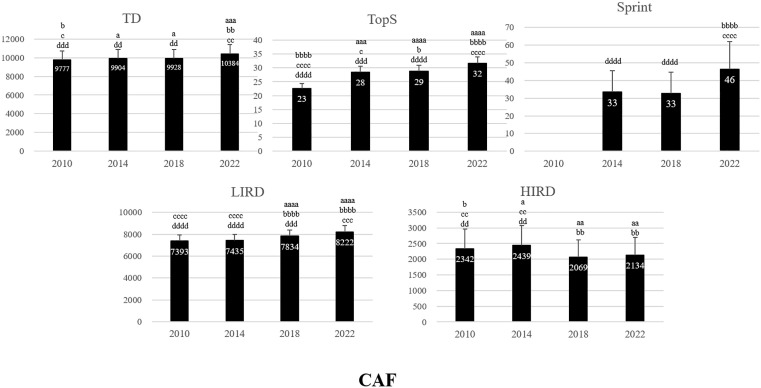
Changes in the values of physical parameters for outfield players in CFA across the 2010–2022 FIFA World Cup. Superscript letters “a–d” denote statistically significant differences relative to the 19th, 20th, 21st, and 22nd cohorts, respectively. The number of letters (1–4) indicates the level of significance: one letter, possible; two letters, likely; three letters, very likely. four letters, most likely.

**Fig 3:**
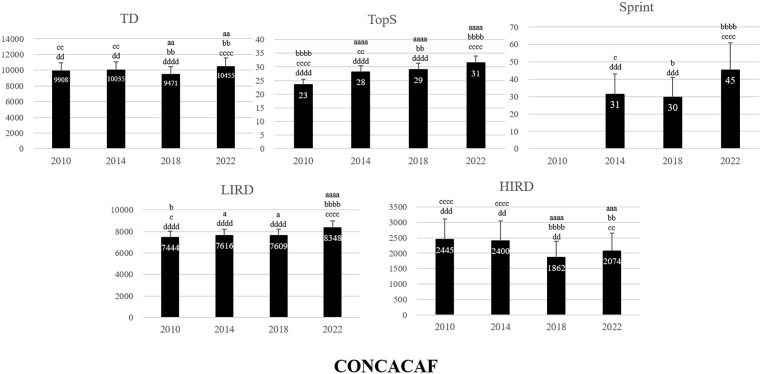
Changes in the values of physical parameters for outfield players in CONCACFA across the 2010–2022 FIFA World Cup. Superscript letters “a–d” denote statistically significant differences relative to the 19th, 20th, 21st, and 22nd cohorts, respectively. The number of letters (1–4) indicates the level of significance: one letter, possible; two letters, likely; three letters, very likely. four letters, most likely.

**Fig 4:**
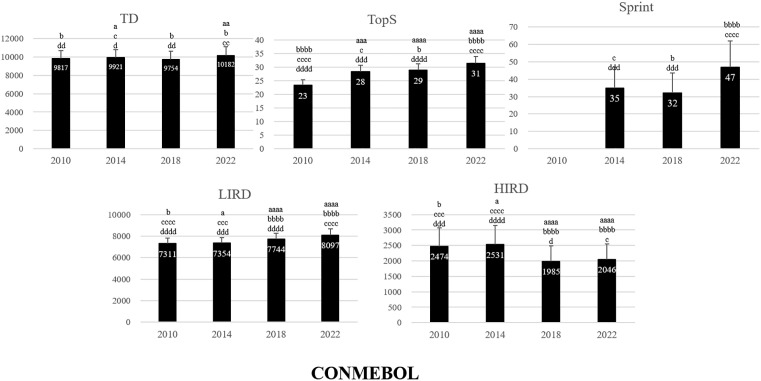
Changes in the values of physical parameters for outfield players in CONMEBOL across the 2010–2022 FIFA World Cup. Superscript letters “a–d” denote statistically significant differences relative to the 19th, 20th, 21st, and 22nd cohorts, respectively. The number of letters (1–4) indicates the level of significance: one letter, possible; two letters, likely; three letters, very likely. four letters, most likely.

**Fig 5:**
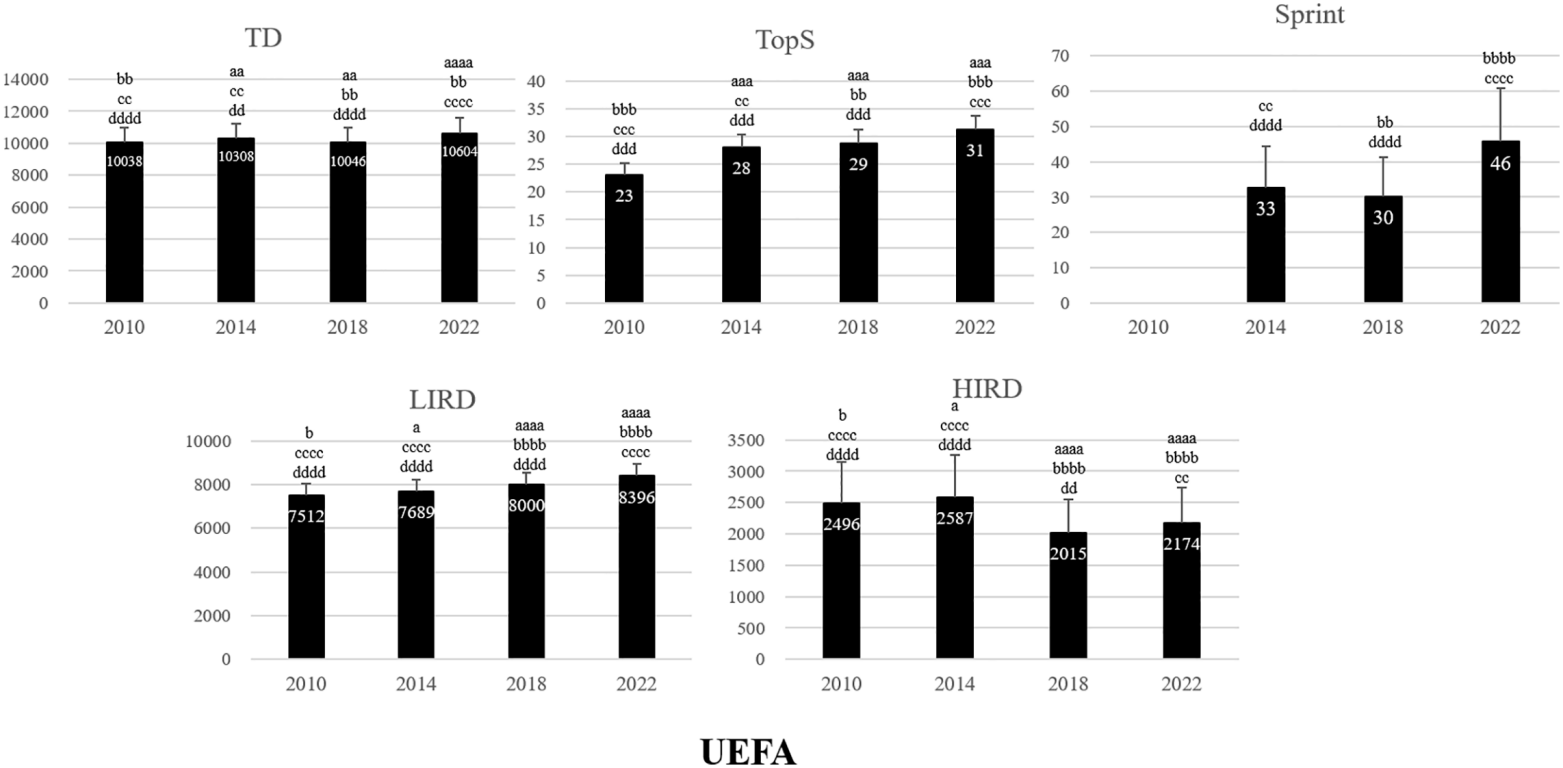
Changes in the values of physical parameters for outfield players in UEFA across the 2010–2022 FIFA World Cup. Superscript letters “a–d” denote statistically significant differences relative to the 19th, 20th, 21st, and 22nd cohorts, respectively. The number of letters (1–4) indicates the level of significance: one letter, possible; two letters, likely; three letters, very likely. four letters, most likely.

Values of the five physical performance parameters for outfield players from the five confederations across the FIFA World Cups (2010–2022) are presented in Figs 1–5. Clear increasing trends in TopS, sprint distance, and LIRD were observed in all confederations. By contrast, the patterns in total distance TD and HIRD differed among confederations. As shown in Figs 1, 3 and 5, TD increased substantially from 2018 to 2022 in AFC, CONCACAF and UEFA, whereas TD showed only trivial changes across editions in CONMEBOL, as illustrated in Fig 4. Furthermore, Figs 1, 4 and 5 demonstrate that HIRD in AFC, CONMEBOL and UEFA was substantially lower in 2018 and 2022 than in 2010 and 2014, while changes in HIRD in CAF remained trivial, as indicated in Fig 2.

## Disscussion

The aim of this study was to examine whether physical performance trends at the FIFA World Cup from 2010 to 2022 followed similar or divergent trajectories across the five confederations. The main findings were that all confederations showed clear increases in top speed (TopS), sprint actions and low-intensity running distance (LIRD) across the four editions, whereas total distance (TD) and high-intensity running distance (HIRD) evolved differently between confederations.

The simultaneous increases in TopS, Sprint and LIRD across all confederations are consistent with evidence that elite football has become faster and more explosive over the past decade. In the English Premier League, Barnes et al. reported ~30–35% increases in high-intensity and sprint distances over seven seasons, with only small changes in TD [17]. Similar trends have been observed in other professional leagues, where high-intensity and sprint running have increased while overall distance remained stable or changed only modestly [18]. Contextualised match analyses in elite competitions have described football as a sequence of frequent high-intensity efforts interspersed with extended periods of low-intensity movement and positional adjustment [19]. Recent work around the Qatar 2022 World Cup likewise emphasised high volumes of very-high-speed and sprint running as a defining feature of contemporary international play [20]. In this context, the present finding that all five confederations increased both maximum speeds and sprint involvement, alongside larger LIRD, indicates that World Cup matches have become faster and more dynamic, with more frequent explosive actions supported by a greater amount of low-intensity running.

The combination of higher TopS and more sprints with greater LIRD suggests a shift in the structure of running load rather than a simple, uniform increase across all speeds. League-based longitudinal studies have shown that while players now perform more actions at very high speeds, the proportion of total distance covered at lower intensities remains substantial [17]. Tuo, Wang [10]reported that in the 2018 World Cup approximately two-thirds of match distance was accumulated at walking or very low speeds, with only a small fraction above 15 km/h. The present results, showing concurrent increases in LIRD and sprint activity, fit this profile: players are required both to execute more high-speed actions and to maintain a larger base of low-intensity running for recovery, repositioning and tactical reorganisation. This pattern aligns with recent descriptions of tournament football that highlight more frequent transitions, faster ball circulation and higher demands on repeated sprint ability across competitions [19].

The evolution of TD was less uniform. In the present data, AFC, CONCACAF and UEFA showed substantial increases in TD from 2018 to 2022, whereas TD in CONMEBOL remained relatively stable across the four editions. Studies from Russia 2018 indicate that UEFA and CONMEBOL teams generally covered more total distance than teams from AFC, CAF and CONCACAF, with a large share of this distance at low intensities [10]. Work comparing 2018 and 2022 editions has reported increases in TD and high-speed distances in some samples, particularly for teams adopting aggressive pressing and transition-oriented approaches [21]. In parallel, studies describing the evolution of match demands in the Premier League and other European contexts have shown that overall distance tends to fluctuate within a relatively narrow range, while intensity distribution changes within that volume [17]. The present confederation-level data suggest that some regions—here, AFC, CONCACAF and UEFA—have moved towards both faster and more voluminous match running at World Cup level, whereas others, such as CONMEBOL, have adapted primarily through changes in speed and intensity distribution without large shifts in TD.

HIRD showed the clearest divergence between confederations. In AFC, CONMEBOL and UEFA, HIRD in 2018 and 2022 was lower than in 2010 and 2014, while CAF displayed only small changes, even though Sprint increased across the same period. Tuo et al. reported that in 2018 only about 7–8% of match distance was covered above 15 km/h, with large between-confederation differences in how low-intensity distance was accumulated [10]. FIFA’s physical reports on Qatar 2022 similarly emphasised that distances at ≥20 and ≥25 km/h are critical for performance but represent a small proportion of the overall load [22]. League-level studies have also shown that increases in high-intensity and sprint distances often occur without proportional rises in broader high-intensity bands, particularly when coaches manage physical load through tactical compactness and more efficient positional structures [23]. Within this wider evidence base, the present observation that sprint frequency increased while HIRD did not increase—and in some cases decreased—suggests a redistribution of effort within the ≥ 15 km/h domain, with more emphasis on short, very fast actions rather than extended high-intensity running.

Considering confederation differences, the present longitudinal results complement cross-sectional studies from recent World Cups. For 2018, Tuo et al. reported that UEFA and CONMEBOL players had similar running profiles, whereas players from AFC, CAF and CONCACAF covered less TD and less low-speed distance but achieved comparable high-intensity and sprint metrics [10]. Other analyses of the same tournament and of Qatar 2022 have shown that European and South American teams tend to combine higher physical outputs with more possession-based or offensively oriented playing styles, while teams from other confederations display different physical–tactical profiles [24]. Recent work benchmarking match performance across men’s and women’s World Cups also indicates systematic confederation differences in running metrics at the most recent tournaments [25]. Against this background, the present data show that, over four World Cup cycles, all confederations have shifted towards higher peak speeds, more sprint actions and greater LIRD, but their trajectories for TD and HIRD have not converged to the same extent. This pattern is consistent with the idea that the global tempo of World Cup football is rising across regions, while regional contexts, tactical cultures and squad profiles continue to shape how total and high-intensity running are organised within matches.

## Conclusion

We concluded that physical performance trends at the FIFA World Cup are only partially consistent across the five confederations. All confederations showed clear increases in TopS, Sprint and LIRD from 2010 to 2022, indicating a shared global shift towards faster and more dynamic match play. In contrast, TD and HIRD followed confederation-specific trajectories: AFC, CONCACAF and UEFA displayed substantial increases in TD, whereas CONMEBOL and CAF showed stable or reduced HIRD despite higher sprint activity, suggesting different ways of organising overall and high-speed loads. Taken together, these findings suggest that coaches and performance staff should consider both the global increase in speed-related demands and the enduring confederation-specific patterns of running performance when benchmarking and preparing teams for World Cup competition.

We also acknowledge several limitations. First, the analysis was based on individual tracking data but did not differentiate playing positions or tactical roles, nor incorporate technical–tactical variables, limiting the ability to relate confederation-level trends to positional demands or game models. Second, running speeds were collapsed into two broad bands (<15 km/h and ≥15 km/h) to harmonise systems across tournaments, improving comparability but reducing intensity resolution. Third, the sample included only outfield players from four men’s World Cups, so the findings may not generalise to qualifiers, other competitions or women’s football. Moreover, teams from the Oceania Football Confederation (OFC) were excluded due to insufficient match sample size, limiting applicability to that confederation. Future research should incorporate positional, technical–tactical and contextual information and draw on broader samples to better understand how confederation-specific physical trends develop.

## Supporting information

S1 Data Original dataset used for statistical analysis. (XLSX)
